# Enhanced Alcohol Preference and Anxiolytic Alcohol Effects in Niemann-Pick Disease Model in Mice

**DOI:** 10.3389/fneur.2019.00731

**Published:** 2019-07-03

**Authors:** Liubov S. Kalinichenko, Christiane Mühle, Volker Eulenburg, Marc Praetner, Martin Reichel, Erich Gulbins, Johannes Kornhuber, Christian P. Müller

**Affiliations:** ^1^Department of Psychiatry and Psychotherapy, University Clinic, Friedrich-Alexander-University of Erlangen-Nuremberg, Erlangen, Germany; ^2^Institute for Biochemistry and Molecular Medicine, Friedrich-Alexander-University Erlangen-Nuremberg, Erlangen, Germany; ^3^Department of Anaesthesiology and Intensive Care Medicine, University of Leipzig, Leipzig, Germany; ^4^Department of Nephrology and Medical Intensive Care, Charité - Universitätsmedizin Berlin, Berlin, Germany; ^5^Department of Molecular Biology, University of Duisburg-Essen, Essen, Germany; ^6^Department of Surgery, College of Medicine, University of Cincinnati, Cincinnati, OH, United States

**Keywords:** acid sphingomyelinase, alcohol, anxiety, depression, stress

## Abstract

Major depression and alcohol use disorder are severe psychiatric diseases affecting the world's population with high comorbidity level. However, the pathogenesis of this comorbidity remains unclear, and no selective treatment for this condition is available. A pathogenic pathway and a possible therapeutic target for the treatment of depression-alcoholism comorbidity based on the hyperfunction of acid sphingomyelinase (Asm) were recently suggested. Here we analyzed the effects of alcohol on the depression/anxiety state of homozygous Asm-knockout mice (Asm − /−), which can be considered as a model of an early stage of Niemann-Pick disease, as well as their drinking pattern under normal and stress conditions. It was observed that forced treatment with alcohol (2 g/kg, i.p.) reduces the anxiety level of Asm−/− mice as measured in the elevated plus maze (EPM) test, but enhances the depression level in the forced swim test (FST). The analysis of drinking pattern of these animals in a free-choice alcohol drinking paradigm revealed higher alcohol intake and preference in Asm−/− mice compared to wild type (wt) littermates. However, this difference was overwritten by the stress exposure. Stronger sedating effects of alcohol were observed in Asm−/− mice compared to wt animals in the loss of righting reflex test after single and repeated alcohol injections (3 g/kg, i.p.). Altogether, the present findings might indicate an Asm involvement in the mechanisms of comorbidity between alcoholism and anxiety/depression.

## Introduction

Alcoholism is a chronic relapsing brain disorder with high medical and social importance (1, 2). In accordance to WHO data from 2016, more than 2.3 billion people worldwide, which refers to more than one third of the population, are current drinkers of alcohol (3). Epidemiological studies indicate the significance of the problem of alcohol dependence and abuse, as globally an estimated 237 million men and 46 million women have alcohol use disorders leading to about 3 millions yearly deaths worldwide (3).

The harmful effects of alcohol are one of the leading risk factors for mental diseases. Alcohol use disorders increase the risk for depression development by a factor of almost five (4). About 80% of patients diagnosed with alcohol dependence develop depression at some periods of their lives (5). On the other hand, alcohol dependence develops in more than 15% of patients with major depressive disorder (5). The time course of alcohol use disorder is aggravated by depression, as this interaction enhances the probability of alcohol relapse in patients with these comorbid disorders (6–9). However, even though the comorbidity between depression and alcohol consumption is of high importance, little is known about the potentially common mechanisms determining this dangerous interaction. Moreover, personalized treatment strategies for these comorbid disorders have not been developed yet.

Recent studies have observed that ceramide, one of the crucial sphingolipids in biological membranes, might be link between depression and alcohol use disorder. Clinical studies showed a significant increase in the level of lysosomal and secretory acid sphingomyelinase (ASM) in peripheral blood cells of patients with acute alcohol intoxication (10–13). Similarly, a rise in the concentrations of total ceramide as well as the products of ceramide metabolism, sphingosine and sphinganine, was observed in the liver of alcohol-fed mice (14).The levels of ceramide precursor sphingomyelin is found to be reduced in the blood serum of rats exposed to liquid alcohol-containing diet as well as in alcohol dependent patients (15). A study by Roux et al (16) showed a significant elevation of ceramide, and especially Cer16:0, level in the brain of rodents exposed to long-term alcohol drinking. Altogether, these data indicate that a significant shift in ceramide pattern occurs in peripheral tissues during alcohol treatment (17, 18).

On another hand, ceramide was shown to be involved in the pathogenesis of depressive disorder. Clinical studies revealed an increase in ASM activity in the peripheral blood mononuclear cells of patients with major depressive disorder, which positively correlated with symptom severity (19). Similarly, post-traumatic stress disorder is associated with elevated ASM activity and ceramide levels in the blood (20). Animal studies have also shown depression-like behavior as well as a reduction in neurogenesis typical for depression in mice with Asm overexpression (tgAsm) (21). Interestingly, several antidepressants from various chemical groups serve as functional inhibitors of ASM (22–24) suggesting that this enzyme might be crucial for the antidepressant effects (25, 26).

In a recent study in mice, paradoxical antidepressant effects of alcohol were found to depend on Asm activity and the regulation of the sphingolipid rheostat in the brain (27, 28). Here we asked how a complete reduction of Asm activity, which is the main mechanism of type A and B Niemann-Pick disease, affects the interaction of depression and alcohol in Asm deficient (Asm − /−) mice. We investigated the behavioral and drinking phenotype of these animals as well as the effects of forced treatment with alcohol on the emotional state.

## Methods

### Animals

Asm−/− mice (*Smpd1*−/−) are characterized by age-dependent sphingomyelin accumulation and serve as a model of type A or B Niemann-Pick disease (29). Male and female Asm−/− mice and respective wild type (wt) littermates (8–12 weeks old) were studied in gender-balanced designs. Animals were housed in groups in standard cages, or individually housed, when the experiment required it. They were provided with food and water *ad libitum*, and kept on a 12:12 h light: dark cycle (lights on at 7.00 am). Behavioral tests were performed during the light cycle between 09:00 and 16:00 h. Room temperature was maintained between 19 and 22°C at a humidity of 55% (±10%). All experiments were carried out in accordance with the National Institutes of Health guidelines for the humane treatment of animals and the European Communities Council Directive (86/609/EEC) and were approved by the local governmental commission for animal health (Regierung von Unterfranken, Peterplatz 9, 97070 Würzburg). The analysis of data was performed by an investigator blind to the genotype of the tested animals.

### Free-Choice Alcohol Drinking Paradigm

Alcohol drinking was tested in naïve 8–9-weeks-old Asm−/− (*n* = 15) and wt (*n* = 16) mice using a two-bottle free-choice drinking paradigm. Each cage was equipped with two constantly available bottles, one of which contained tap water and another bottle contained alcohol (Carl Roth). After an acclimatization period to establish a water-drinking baseline, animals received alcohol at increasing concentrations of 2, 4, 8, 12, and 16 vol. %. Mice were exposed to each concentration of alcohol for 4 days. The bottles were weighed and their positions were changed every second day. Thereafter, alcohol concentration was maintained at 16 vol. % for a total of 13 days. At day 9 of the exposure to 16 vol. % alcohol the animals were exposed to 3-days' forced swim stress. For this purpose, each mouse was placed into a glass transparent cylinder (17 cm diameter, 18 cm height) filled with water (12 cm, 25°C) for 15 min. The consumed amount of alcohol relative to body weight and the preference vs. water were measured and corrected for fluid loss (27, 30).

### Taste Preference Test

Alcohol experienced Asm−/− animals (29 days of exposure to free-choice drinking, as described above) were used for this test. Sucrose (0.5 and 5%; Merc Chemicals) and quinine (2 and 20 mg/dl; Merc Chemicals) preference was measured in a two-bottle free-choice test vs. water, 3 days after the last alcohol exposure. Each dose was offered for 2 days with the position of the bottles being changed and weighed daily with 1 day wash out between sucrose and quinine testing (27, 30).

### Loss of Righting Reflex

Alcohol naïve 8-weeks-old animals (Asm−/−, *n* = 23; wt, *n* = 21) were used for this test. Animals were administered with an alcohol injection of 3.5 g/kg (i.p., 20 ml/kg) for 7 consecutive days at the same time. Testing of loss of righting reflex (LORR) was performed at days 1 and 8 of the treatment. After an alcohol injection, the animals were immediately placed in an empty cage. LORR was observed when the animals became ataxic and stopped moving for at least 30 s. Each animal was then placed on its back. Recovery from LORR was defined as the animal being able to right itself three times within a minute. Time taken for the animals to lose its righting reflex, and time to recovery from the alcohol effect was recorded (27, 30).

### Blood Alcohol Concentration

Alcohol naïve 8–10-weeks-old animals (Asm−/−, *n* = 8; wt, *n* = 7) were used for this test. Mice received alcohol injections (3 g/kg, 20 ml/kg, i.p.) and 20 μl blood samples were obtained from the submandibular vein 1, 2, and 3 h after alcohol injection. The blood samples were directly mixed with 80 μl 6.25 % (w/v) trichloroacetic acid (Sigma). After centrifugation 15 μl of the supernatant were subjected to enzymatic alcohol determination using the alcohol dehydrogenase method as described elsewhere (31, 32).

### Behavioral Testing

Alcohol naïve 8–10-week-old mice were tested in a battery of behavioral tests in the following order: open field (OF), elevated plus maze (EPM), novelty suppressed feeding (NSF), and forced swim test (FST). Animals received injections of alcohol (2 g/kg, 10 ml/kg; i.p.; Carl Roth) or physiological saline 30 min before each test. Group sizes were the same for all tests (Asm−/− receiving alcohol: *n* = 9; Asm−/− receiving saline: *n* = 8; wt receiving alcohol: *n* = 14; wt receiving saline: *n* = 12). All tests were performed on separate days between 09:00 and 15:00 h with at least 2 days' interval between them. Mice were moved to the behavioral test room 1 h before testing and were tested in a pseudorandom order. Each test apparatus was cleaned with 70% alcohol between subjects.

#### Open Field

Each mouse was placed in a square white acrylic arena (50 × 50 cm), facing a wall, for 20 min and allowed to freely explore the arena. The area was lighted with white light (100 lx) (33). Video recordings were taken and analyzed using Biobserve Viewer III (Biobserve GmbH, Germany). A virtual square of equal distance from the periphery (25 × 25 cm) was defined as the “central zone,” and the outer part of the arena was defined as “peripheral zone.” Distance moved in the peripheral and central zones, number of entries, latent period of the first entrance, and time spent in the central zone were registered (27, 34).

#### Elevated Plus Maze

The EPM (35) was constructed from black opaque acrylic with white lining on the floor, each arm measuring 30 × 5 cm and the central platform 5 × 5 cm. Two opposite arms were enclosed by a 15-cm wall of opaque acrylic, while the other two arms were open with a ledge of 0.5 cm on the sides. The maze was elevated 50 cm from the ground. Each mouse was placed on the central platform, facing toward a closed arm, and allowed to freely explore the maze for 5 min. Biobserve Viewer III tracking software (Biobserve GmbH, Germany) was used to record the distance moved in the open and closed arms, and the number of entries into the closed and open arms and time spent in them (27, 34).

#### Novelty Suppressed Feeding

Animals were deprived from food for 24 h before the test. After deprivation each mouse was put in the corner of a square white acrylic arena (50 × 50 × 50 cm, 100 lx), facing a wall. A piece of food was placed in the center of the arena. Video recordings were taken and analyzed using Biobserve Viewer III (Biobserve GmbH, Germany). The time (s) before a mouse began eating after placing to the arena and the distance moved before eating were registered (27, 34).

#### Forced Swim Test

At the day 1 of the test, each mouse was placed into a glass transparent cylinder (18 cm diameter, 19 cm height) filled with water (13 cm, 25°C) for 15 min (36). After 24 h mice were again placed in this cylinder with water for 5 min. The latency of first floating, and total floating time during the day 2 were recorded (Biobserve Viewer III, Biobserve GmbH, Germany) and analyzed manually (27, 34).

### Enzyme Activity Measurement

Asm activity was measured in the dorsal hippocampus (DH) of Asm−/− mice after forced treatment with alcohol and following behavioral testing. In addition, the activity of Asm, neutral sphingomyelinase (Nsm), neutral ceramidase (Nc), and acid ceramidase (Ac) were measured in the DH of Asm−/− mice exposed to alcohol treatment on the model of two-bottle free-choice alcohol drinking. After the behavioral testing the animals were sacrificed, brain tissue was harvested and snap frozen with dry ice. The DH was isolated from the brains for enzyme analysis.

The Asm and Nsm activity was determined using the fluorescent substrate BODIPY-FL-C12-SM (N-(4,4-difluoro-5,7-dimethyl-4-bora-3a,4adiaza-s-indacene-3-dodecanoyl)sphingo-syl phosphocholine, Invitrogen/Life Technologies), with four replicates for each sample. NBD-C12-Cer fluorescent substrate was used for the analysis of Ac and Nc activities. Tissues were homogenized in lysis buffer (250 mM sucrose, 1 mM EDTA, 0.2% Triton X-100, 1 × Roche protease inhibitor cocktail, 400 μl/10 mg tissue) using a TissueLyser LT bead mill (Qiagen) with steal beads followed by freezing at −80°C to enhance lysis. Supernatants obtained after centrifugation at 13,000 × g and 4°C for 10 min were diluted 1:3 in lysis buffer and used for activity assays and for protein determination (Bradford/Coomassie kit, ThermoFisher). A standard enzyme reaction contained 58 pmol BODIPY-FL-C12-SM (for Asm and Nsm) or 50 pmol NBD-C12-Cer (for Ac and Nc) as a substrate and 2 μl tissue lysate in a total volume of 50 μl reaction buffer of the following composition: 200 mM sodium acetate buffer (pH 5.0), 500 mM NaCl, 0.2% Nonidet P-40 detergent for Asm, 200 mM HEPES buffer (pH 7.0), 200 mM MgCl_2_, 0.05% Nonidet P-40 for Nsm; 200 mM sodium acetate buffer (pH 4.5), 100 mM NaCl, 0.03% Nonidet P-40 for Ac and 200 mM HEPES (pH 7.0), 100 mM NaCl, 0.03% Nonidet P-40 for Nc. Further analysis using thin layer chromatography was performed as described previously (27, 37).

### Statistical Analysis

All data are presented as means ± standard error of the mean (S.E.M.). The data were compared using a two-way analysis of variance (ANOVA; IBM SPSS Statistics 21). Following a significant ANOVA, the independent pre-planned comparisons were made. Since the specific scientific hypotheses was investigated and all comparisons among means were considered to be of substantive interest a priori, the Fisher's LSD test was used for pre-planned comparisons between individual groups. Pair-wise comparisons using two tailed *t*-tests (IBM SPSS Statistics 21) were performed between trials for each genotype group (38). Although sex differences are well-known in the studies on addiction and depression (39, 40), we did not see significant sex differences in the major parameters of this study, and thus the data were collapsed for analysis. A significance level of *p* < 0.05 was used for statistical significance.

## Results

### Free-Choice Alcohol Drinking

**Figure 1:**
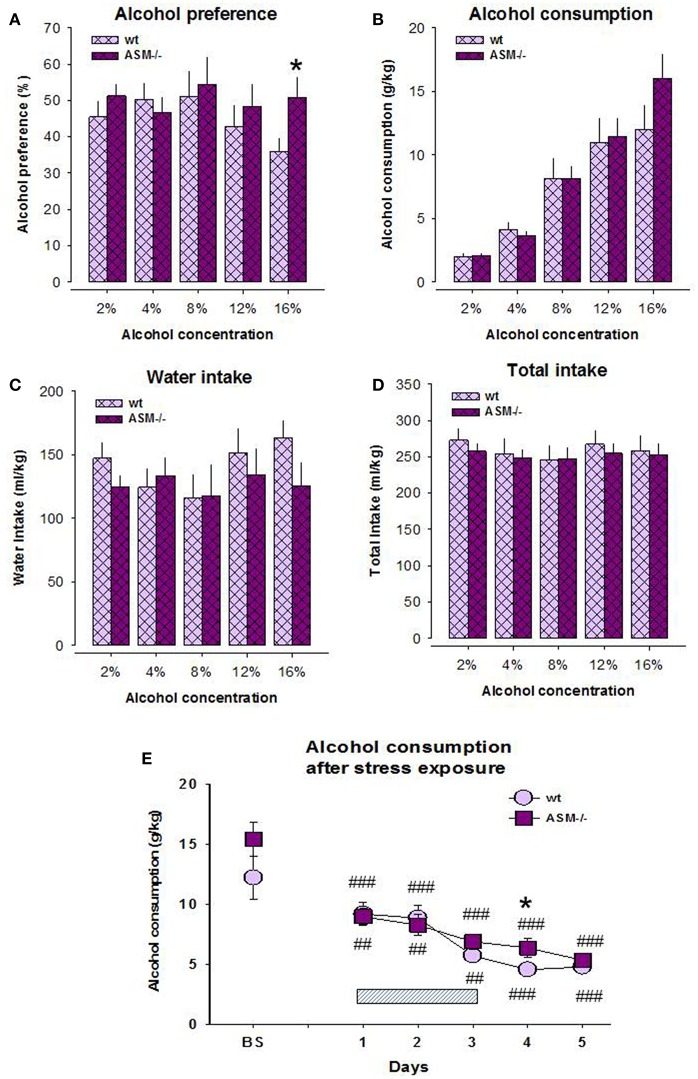
Alcohol preference **(A)** and alcohol consumption **(B)** on the model of two-bottle free-choice drinking is enhanced in homozygous mice with acid sphingomyelinase deficiency (ASM−/−) compared to wild type animals (wt). The levels of water intake **(C)** and total fluid intake **(D)** do not significantly differ between ASM−/− and wt mice. Stress **(E)** overwrites the differences in the drinking pattern of these animals. The hatched box indicates the time of stress exposure. The error bars show the mean ± SEM (*p < 0.05 vs. wt animals; ###*p* < 0.001, ##*p* < 0.01 vs. baseline, BS).

Previous data indicate the contribution of Asm in alcohol sensitivity and pathogenesis of alcohol use disorder (10, 11, 14, 15, 27). Here, we report the higher preference of alcohol on the model of free-choice alcohol drinking in Asm−/− mice compared to wt littermates. A two-way ANOVA for alcohol preference did not reveal a significant effect for genotype [*F*_(1, 14)_ = 0.659, *p* = 0.431] and dose [*F*_(4, 56)_ = 2.408, *p* = 0.060], as well as interaction of these factors [*F*_(4, 56)_ = 2.114, *p* = 0.091; Figure 1A]. However, the pre-planned comparison showed a significantly higher alcohol preference at 16 vol. % alcohol in Asm−/− mice compared to wt animals (*p* = 0.042). The ANOVA analysis for alcohol consumption also did not show a genotype effect [*F*_(1, 14)_ = 0.326, *p* = 0.577], but revealed a dose effect [*F*_(1, 14)_ = 81.809, *p* < 0.001]. A genotype × dose interaction for alcohol consumption was not significant [*F*_(1, 14) =_ 1.583, *p* = 0.229; Figure 1B]. Single day comparison showed slightly higher alcohol consumption at 16 vol. % alcohol concentration in Asm−/− mice, which, however, did not reach statistical significance (*p* = 0.160). A two-way ANOVA for total fluid intake showed no significant effect for the genotype [*F*_(1, 14)_ = 0.107, *p* = 0.748], but a significant dose effect [*F*_(4, 56)_ = 4.443, *p* = 0.003; Figure 1D]. A genotype × dose interaction for this parameter was not significant [*F*_(4, 56)_ = 0.838, *p* = 0.507]. Similarly, ANOVA for water intake revealed no significant effect for the genotype [*F*_(1, 14)_ = 0.401, *p* = 0.537] or genotype × dose interaction [*F*_(4, 56)_ = 2.394, *p* = 0.061], but a significant dose effect [*F*_(4, 56)_ = 3.356, *p* = 0.016; Figure 1C]. However, pre-planned comparison did not reveal significant differences between genotypes at various alcohol concentrations (*p* > 0.05).

The analysis of alcohol drinking after 3-days' swim stress showed no genotype effect [*F*_(1, 14)_ = 1.626, *p* = 1.223], but a significant effect of stress [*F*_(5, 70)_ = 31.697, *p* < 0.001; Figure 1E]. The genotype × stress interaction was not significant [*F*_(5, 70)_ = 1.475, *p* = 0.209]. Pre-planned comparison revealed a significantly lower post-stress alcohol consumption both in Asm−/− and wt mice at all test days, respectively (day 1: *p* < 0.001, *p* = 0.025; day 2: *p* < 0.001, *p* = 0.035; day 3: *p* < 0.001, *p* = 0.002; day 4: *p* < 0.001, *p* < 0.001; day 5: *p* < 0.001, *p* < 0.001). Alcohol consumption did not differ between Asm−/− and wt mice during and after stress except for the first post-stress day (*p* = 0.041). These data suggest that a complete lack of Asm is associated with the higher susceptibility of mice to alcohol consumption at high doses. This effect is overwritten by stress exposure.

### Taste Preference

**Figure 2:**
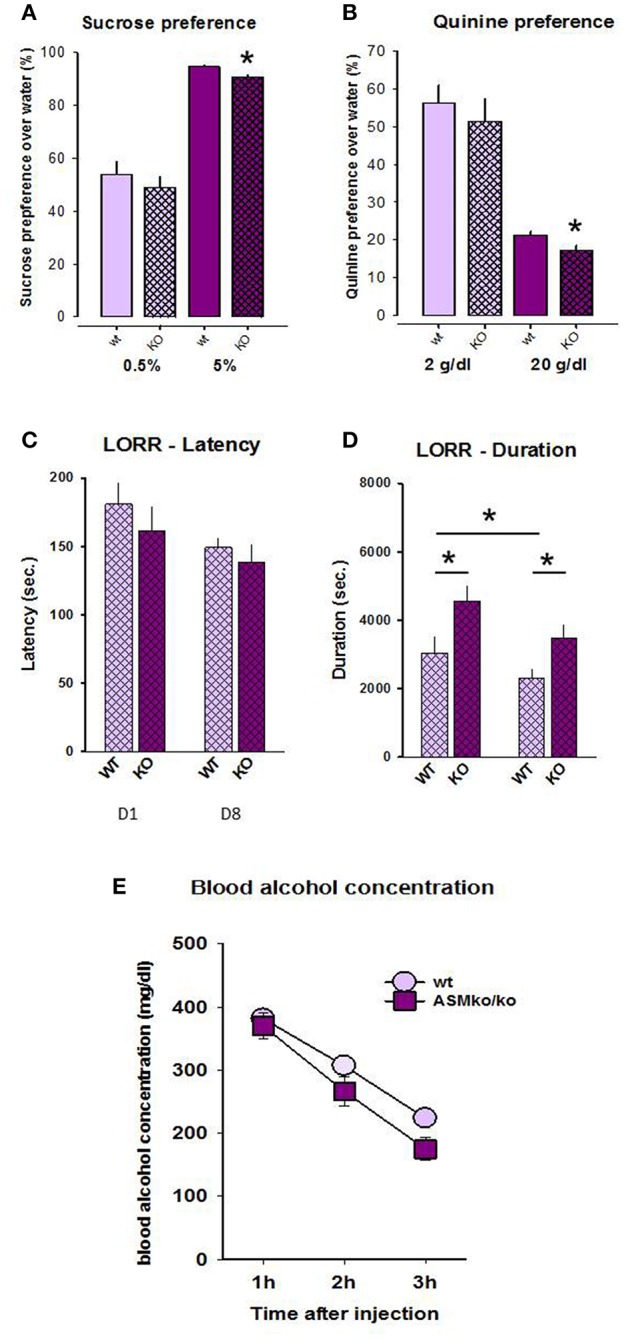
Sucrose **(A)** and quinine **(B)** preference is lower in mice with acid sphingomyelinase deficiency (KO) compared to wt littermates. The duration **(D)**, but not the latency **(C)** of loss of righting reflex is significantly higher in mice with acid sphingomyelinase deficiency both at day 1 and 8 of alcohol treatment. Blood alcohol concentration **(E)** does not significantly differ between mice with acid sphingomyelinase deficiency and wt mice. The error bars show the mean ± SEM (*p < 0.05 vs. wt animals).

Statistical analysis did not reveal any genotype differences in the preference of 0.5% sucrose (*t* = −0.741, *p* = 0.471; Figure 2A). However, the preference of 5% sucrose was significantly higher in wt mice compared to Asm−/− animals (*t* = −3.168, *p* = 0.007). Similarly, no differences were observed in the preference of quinine in the dose of 2 mg/dl (*t* = −0.647, *p* = 0.529), but the preference of quinine in the dose of 20 mg/dl was higher in wt mice (*t* = −2.450, *p* = 0.030; Figure 2B). These data indicate that a complete lack of Asm mildly reduces hedonic preference, but enhances avoidance of aversive stimuli.

### Loss of Righting Reflex

No significant genotype [*F*_(1, 36)_ = 2.298, *p* = 0.138] or time effect [*F*_(1, 36)_ = 2.918, *p* = 0.096], as well as no genotype × time interaction [*F*_(1, 36)_ = 0.229, *p* = 0.635] were observed for LORR latency (Figure 2C). Pre-planned comparison did also not yield genotype-specific and time-induced differences in this parameter (*p* > 0.05).

A two-way ANOVA for the duration of LORR revealed a genotype effect [*F*_(1, 22)_ = 12.609, *p* = 0.002] as well as time effect [*F*_(1, 22)_ = 9.936, *p* = 0.005], but no genotype × time interaction [*F*_(1, 22)_ = 1.073, *p* = 0.312; Figure 2D]. Pre-planned comparison showed that the duration of LORR is significantly higher in Asm−/− mice compared to wt animals both at day 1 (*p* = 0.016) and 8 (*p* = 0.016). The duration of LORR in wt animals significantly declined from day 1 to day 8 (*p* = 0.015). This effect was also observed in Asm−/− mice, but did not reach statistical significance (*p* = 0.098). These findings suggest that a complete lack of Asm makes animals more susceptible to sedative effects of alcohol.

### Blood Alcohol Concentration

A two-way ANOVA for blood alcohol concentration revealed a genotype effect [*F*_(1, 12)_ = 5.551, *p* = 0.036] as well as time effect [*F*_(2, 24)_ = 44.537, *p* < 0.001], but no genotype × time interaction [*F*_(2, 24)_ = 0.544, *p* = 0.587; Figure 2E]. Pre-planned comparison did not show significant genotype differences in the blood alcohol concentration within 1 h (*p* = 0.671), 2 h (*p* = 0.165), but a tendency after 3 h (*p* = 0.057) after the injection. These finding suggests a preserved bioavailability of alcohol in Asm−/− mice.

### The Activity of Enzymes Mediating Ceramide Metabolism After Free-Choice Alcohol Drinking

**Figure 3:**
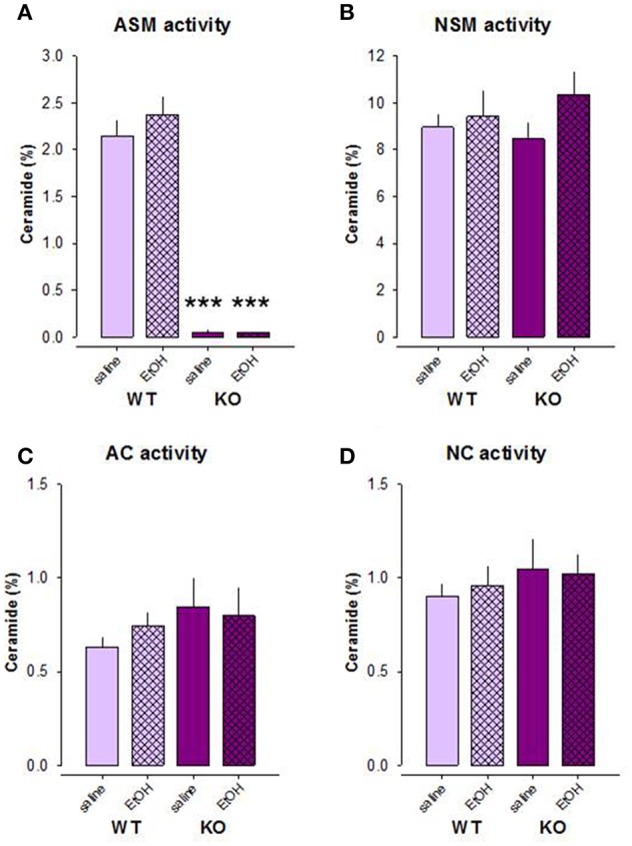
The activity of Asm **(A)** is significantly lower in the dorsal hippocampus of mice with acid sphingomyelinase deficiency (KO) exposed and not exposed to alcohol treatment on the model of two-bottle free-choice drinking. No significant differences in the activities of neutral sphingomyelinase **(B)**, acid ceramidase **(C)**, and neutral ceramidase **(D)** are observed under these conditions. The error bars show the mean ± SEM (***p < 0.001 vs. wt animals).

Our previous data indicate that the effects of alcohol on the depression/anxiety-like behavior of mice are mediated by the alterations in brain Asm activity as well as the state of sphingolipid rheostat (27). Here we showed that other enzymes of the sphingomyelinase pathway are not involved in the effects of alcohol. A two-way ANOVA revealed a strong genotype effect for Asm activity in the DH of mice exposed to the free-choice alcohol drinking paradigm [*F*_(1, 37)_ = 215.091, *p* < 0.001], but no treatment effect [*F*_(1, 37)_ = 0.549, *p* = 0.463] or genotype × treatment interaction [*F*_(1, 37)_ = 0.590, *p* = 0.447; Figure 3A]. Pre-planned comparison did also not reveal alcohol effects on the Asm activity in Asm−/− (*p* = 0.986) or wt mice (*p* = 0.248). As expected, Asm activity was significantly lower in Asm−/− mice drinking both water and alcohol compared to wt mice (*p* < 0.001).

No significant genotype [*F*_(1, 37)_ = 0.062, *p* = 0.805] or treatment effects [*F*_(1, 37)_ = 1.770, *p* = 0.192] on Nsm activity or a genotype × treatment interaction [*F*_(1, 37)_ = 0.659, *p* = 0.422] were observed (Figure 3B). Nsm activity did not differ between Asm−/− and wt mice receiving water (*p* = 0.697) or alcohol (*p* = 0.450). Moreover, alcohol had no effect on this parameter in wt (*p* = 0.689) and Asm−/− (*p* = 0.170) mice.

Similarly, the activity of Ac and Nc was also not altered by alcohol treatment in Asm−/− or wt mice (Figures 3C,D). A two-way ANOVA did not reveal an effect of genotype [Ac: *F*_(1, 37)_ = 1.658, *p* = 0.206; Nc: *F*_(1, 37)_ = 0.953, *p* = 0.335] or the treatment [Ac: *F*_(1, 37)_ = 0.105, *p* = 0.747; Nc: *F*_(1, 37)_ = 0.025, *p* = 0.876], as well as no effects of genotype × treatment interaction [Ac: *F*_(1, 37)_ = 0.597, *p* = 0.444; Nc: *F*_(1, 37)_ = 0.150, *p* = 0.701]. Pre-planned comparison also did not reveal any alcohol-induced alterations in the activity of Nc or Ac in the animals (*p* > 0.05). Altogether, these findings confirm the efficiency of Asm−/− model and a lack of counteracting mechanisms in the enzymes of the ceramide rheostat. They also show that alcohol consumption has no effects on Asm, Nsm, Ac, and Nc activity in the DH of animals.

### Forced Treatment With Alcohol

#### Elevated Plus Maze

**Figure 4:**
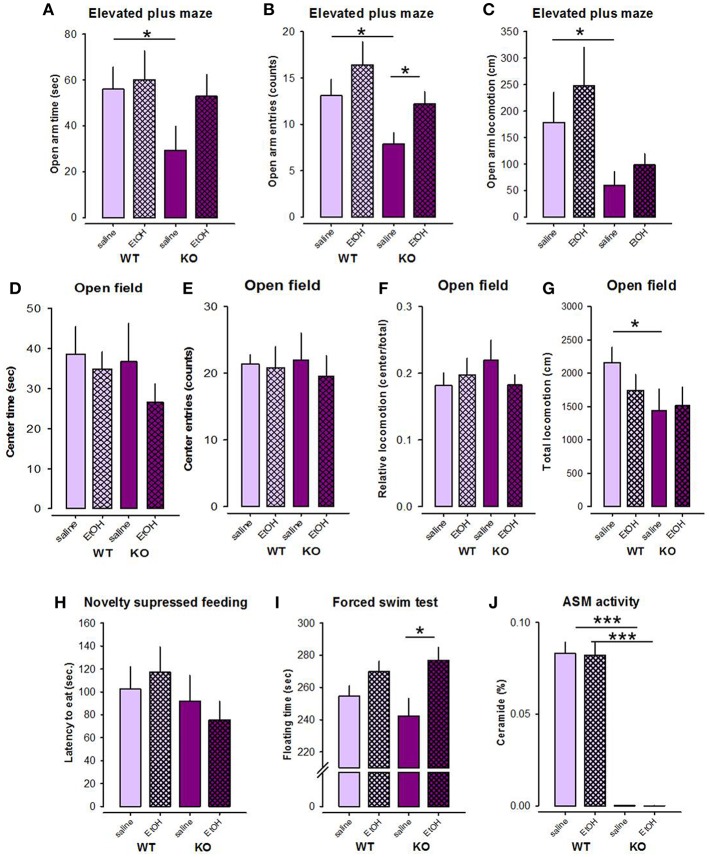
Forced treatment with alcohol modeled by intraperitoneal injection with alcohol reduces anxiety level selectively in mice with acid sphingomyelinase deficiency (KO) as observed in the elevated plus maze **(A–C)**, but not in the open field test **(D–G)**. Alcohol injections do not affect the behavior of mice in the novelty suppressed feeding test **(H)**, but has depressogenic effect in KO mice in the forced swim test **(I)**. Forced treatment with alcohol does not affect acid sphingomyelinase (Asm) activity in the dorsal hippocampus of KO and wild type (wt) mice **(J)**. The error bars show the mean ± SEM (***p < 0.001, *p < 0.05 vs. wt animals).

Previous data showed the alterations in depression/anxiety-like behavior in mice with altered Asm activity (21, 27), which in the animals with Asm overexpression can be specifically altered by alcohol treatment (27). Here we observed higher anxiety level in Asm−/− mice in the EPM test. An ANOVA revealed significant genotype differences (Asm−/− vs. wt) between the number of entries to the open arms and locomotion in the open arms of the EPM [*F*_(1, 37)_ = 5.781, *p* = 0.021; *F*_(1, 37)_ = 6.018, *p* = 0.019], respectively, but not in the time spent in the open arms [*F*_(1, 37)_ = 2.304, *p* = 0.138; Figures 4A–C]. However, no treatment effects [*F*_(1, 37)_ = 3.803, *p* = 0.059; *F*_(1, 37)_ = 0.975, *p* = 0.330; *F*_(1, 37)_ = 1.524, *p* = 0.225] or genotype × treatment interactions [*F*_(1, 37)_ = 0.065, *p* = 0.800; *F*_(1, 37)_ = 0.073, *p* = 0.788; *F*_(1, 37)_ = 0.761, *p* = 0.389] were observed for these parameters. Pre-planned comparisons showed that Asm−/− mice receiving saline were characterized by lower number of entries to the open arms (*p* = 0.014), reduced locomotion in the open arms (*p* = 0.038), and also lower time spent in the open arms (*p* = 0.041) of the EPM compared to wt littermates (Figures 4A–C). These data indicate higher level of anxiety in alcohol-naïve Asm−/− mice. Asm−/− mice receiving alcohol were characterized by the significantly higher number of entries to the open arms comparing to the Asm−/− animals treated with saline (*p* = 0.015). Similar tendencies were found for the time spent in the open arms (*p* = 0.060) and locomotion in the open arms (*p* = 0.128). However, no significant changes in the study parameters were observed in wt animals injected with alcohol (*p* > 0.05). Thus, these data indicate that the high anxiety observed in Asm−/− mice was reversed by the forced alcohol treatment.

#### Open Field

An ANOVA for the time spent in the center of the OF did not reveal significant genotype [*F*_(1, 38)_ = 0.667, *p* = 0.419] or treatment effects [*F*_(1, 38)_ = 1.257, *p* = 0.269], as well as no genotype × treatment interaction [*F*_(1, 38)_ = 0.269, *p* = 0.607; Figure 4D]. No effects of alcohol on this parameter were observed in wt and Asm−/− mice (*p* > 0.05). Similarly, no genotype effect [*F*_(1, 38)_ = 0.010, *p* = 0.921], treatment effect [*F*_(1, 38)_ = 0.231, *p* = 0.634], or genotype × treatment interaction [*F*_(1, 38)_ = 0.096, *p* = 0.758] was found for the analysis of the number of entries to the center of the OF (Figure 4E). Pre-planned comparison did not show any effects of alcohol on the number of entries to the center of the OF in Asm−/− or wt animals (*p* > 0.05). Similarly, the relative locomotion of the study animals was also not altered by alcohol [genotype effect: *F*_(1, 38)_ = 0.244, *p* = 0.624; treatment effect: *F*_(1, 38)_ = 0.202, *p* = 0.656; genotype × treatment interaction: *F*_(1, 38)_ = 1.329, *p* = 0.256; Figure 4F]. Pre-planned comparison did not reveal alcohol-induced changes in the relative locomotion of Asm−/− or wt animals (*p* > 0.05). Total locomotion tended to decrease in Asm−/− mice compared to wt littermates [genotype effect: *F*_(1, 38)_ = 3.170, *p* = 0.083; treatment effects: *F*_(1, 38)_ = 0.436, *p* = 0.513; genotype × treatment interaction: *F*_(1, 38)_ = 0.873, *p* = 0.356; Figure 4G]. Pre-planned comparison confirmed a significant reduction in total locomotion in alcohol-naïve Asm−/− mice receiving vehicle compared to wt mice (*p* = 0.023).

#### Novelty Suppressed Feeding

ANOVA revealed no genotype or treatment effects on the latency of first eating in the NSF test [*F*_(1, 38)_ = 1.599, *p* = 0.214; *F*_(1, 38)_ = 0.002, *p* = 0.969; Figure 4H]. Genotype × treatment interaction was also not significant [*F*_(1, 38)_ = 0.568, *p* = 0.456]. Pre-planned comparisons also did not yield significant between-group differences (*p* > 0.05).

#### Forced Swim Test

An ANOVA did not show a genotype effect [*F*_(1, 38)_ = 0.117, *p* = 0.734], but a significant treatment effect on time of floating in the FST [*F*_(1, 38)_ = 9.907, *p* = 0.003; Figure 4I]. The genotype × treatment interaction was not significant [*F*_(1, 38)_ = 1.508, *p* = 0.227]. Pre-planned comparison showed that alcohol treatment induced a significant increase in floating time in Asm−/− mice (*p* = 0.013), and a tendency to increase in wt animals (*p* = 0.056). These data suggest that forced alcohol administration has depressogenic effect, which are elevated in mice with lack of Asm.

#### Asm Activity After Forced Alcohol Treatment

An ANOVA revealed a significant genotype effect on the Asm activity in the DH [*F*_(1, 39)_ = 299.415, *p* < 0.001], but not treatment effect [*F*_(1, 39)_ = 0.053, *p* = 0.820] or genotype × treatment interaction [*F*_(1, 39)_ = 0.045, *p* = 0.833; Figure 4J]. Asm activity in the DH of Asm−/− mice receiving saline was significantly lower than in wt specimens (*p* < 0.001). Subchronic treatment with alcohol (2 g/kg) did not affect Asm activity in the DH neither in Asm−/− (*p* = 0.213), nor in wt animals (*p* = 0.391). These findings suggest that the forced administration of alcohol does not affect Asm activity in the DH.

## Discussion

Ceramide is an important lipid molecule shown to contribute to the interaction between alcohol use disorder and depression via an Asm-mediated rebalancing of the sphingomyelinase rheostat (27). Here we report that Asm deficiency in mice is associated with enhanced alcohol preference in a two-bottle free-choice drinking paradigm. However, stress exposure overwrites the differences in the drinking phenotype of Asm−/− and wt mice. Forced alcohol treatment modeled by intraperitoneal alcohol injections reverses the high anxiety-like behavior in Asm−/− mice, but does not affect anxiety-related behavior in wt animals. However, acute alcohol treatment induced depression-like behavior specifically in Asm−/− mice. Interestingly, the effects of alcohol on the emotional state of the mice were not mediated by alteration/inhibition of ceramide synthesis. Thus, these data expand the specific involvement of Asm in the interconnection between alcoholism and anxiety/depression in mice.

The analysis of drinking pattern in Asm−/− mice showed a higher alcohol preference on the two-bottle free-choice alcohol drinking compared to wt animals. The total fluid intake did not differ between Asm−/− and wt mice reflecting the specificity of the observed pattern of alcohol consumption. Interestingly, the BAC study has observed slightly reduced alcohol availability 3 h after alcohol administration in Asm−/− mice. This might indicate higher metabolic rate of alcohol in these animals compared to wt mice. These results might possibly explain the higher consumption of alcohol in the free-choice alcohol drinking paradigm in Asm−/− mice. In order to reach the same blood alcohol concentration (BAC) over time, Asm−/− mice may have to consume higher amounts of alcohol compared to wt animals. These data are in line with the data obtained in patients with chronic alcoholism. They show greater tolerance to alcohol due to an increase in the alcohol elimination rate and metabolic tolerance (41). Previous animal studies also confirm this hypothesis as alcohol-preferring rats are characterized by a significantly lower blood alcohol concentration compared to non-preferring controls (42), probably due to more rapid alcohol metabolism and higher activity of alcohol dehydrogenase (43).

The analysis of taste preference in Asm−/− mice showed a slightly lower preference of sucrose and lower avoidance to quinine in these animals compared to wt littermates, which reached significance only at high doses of both substances (20 g/dl and 5%). These data might reflect a higher aversion developing in Asm−/− mice and a reduced hedonic perception. Thus, the higher preference of alcohol in these animals compared to wt mice might indicate the specificity of alcohol drinking phenotype, which is unlikely due to the changes in the hedonic and aversion avoidance.

The exposure to stress on the model of forced swimming induced a decrease in alcohol consumption in wt and Asm−/− mice and overwrote the genotype-specific difference in the alcohol drinking pattern. The consumption of alcohol was reduced after stress in both Asm−/− and wt mice. These data are in line with previous studies. It is well-known that stress is a factor triggering alcohol consumption in humans as well as in animal models. However, human studies have observed ambiguous effects of stress on the pattern of drinking in both females and males. Even though the frequency of heavy drinking has positive correlation with the stress level, the frequency of moderate drinking decreased with an increase in stress levels (44, 45). The model of two-bottle free-choice alcohol drinking allows animals to self-titrate with alcohol and to maintain a moderate level of drinking as confirmed by BAC studies (46). Moreover, our data suggested that alcohol treatment enhances depression level in wt and particularly Asm−/− mice. Taking into account that FST may induce a depressive state (47), it may be suggested that Asm−/− mice reduce alcohol consumption after stress to possibly reduce the depression level.

It was proposed that re-balancing of brain sphingolipid composition after stress exposure might play a protective role against a stress-induced increase in alcohol consumption. Previous studies revealed a significant dysregulation of sphingolipid and phospholipid metabolism in rats after chronic unpredictable stress (48). In the prefrontal cortex, chronic unpredictable stress was followed by an increase in ceramide, lysophosphatidylethanolamine, and 38 carbon (38C)-lipid levels, and a reduction in sphingomyelin and dihydrosphingomyelin concentrations, phosphatidylethanolamine, ether phosphatidylcholine, and 36C-lipid levels. Similarly, a decrease in phosphatidylcholine and sphingomyelin, and an increase in phosphatidylserine, ceramide, and dihydrosphingomyelin levels were observed in the hippocampus of rats exposed to chronic unpredictable stress (48). As it was shown, various lipid species contribute to the development of addiction, and particularly alcohol dependence (18), and thus the reestablishment of lipid balance after stress exposure might determine the absence of increase in alcohol consumption in Asm−/− mice.

The analysis of LORR showed higher sedative effects of alcohol in Asm−/− mice compared to wt animals at day 1 and 8 reflecting the higher sensitivity of the CNS to alcohol. These data are in line with previous studies showing that mice with higher alcohol preference display higher initial sensitivity to alcohol, as measured by the static dowel task (49). Similarly, human studies also suggested a higher sensitivity to the subjective effects of alcohol in patients with a family history of alcohol use disorder (50). Interestingly, the latency and duration of LORR significantly decreased in wt animals and tended to decrease in ASM−/− mice at day 8 of the experiment, reflecting the development of alcohol tolerance.

Interestingly, the observed drinking phenotype of Asm−/− mice resembles the phenotype of mice with Asm overexpression, but not heterozygous Asm knockout mice (Asm+/−) as it was shown in our previous study (27). It might be proposed that the complete knockout of the ASM coding gene induces possible alterations in other pathways, particularly other mechanisms of ceramide synthesis, such as *de novo* pathway, in Asm−/− mice, which may independently contribute to elevated alcohol drinking levels. The *de novo* pathway was shown to mediate the elevation in ceramide synthesis in the liver during alcohol consumption reflecting that both, sphingomyelinase and *de novo* pathways, contribute to the ceramide response to alcohol treatment (51–53). Particularly, Tong et al. (54) showed that an inhibitor of the *de novo* ceramide synthesis, myriocin, inhibits a rise in liver ceramide level and the severity of alcohol-related steatohepatitis in rats exposed to chronic treatment with alcohol. However, single treatment of animals with myriocin does not completely block the observed alcohol-induced changes (51–53). Thus, it might be speculated that the *de novo* pathway of ceramide synthesis determines the similarity in the drinking pattern of Asm−/− mice and animals with Asm overexpression. Moreover, recent data showed that pharmacological inhibition of Asm in mice by antidepressants amitriptyline and fluoxetine is followed by the reduction of lysosomal ceramide level, but the accumulation of ceramide in the endoplasmic reticulum (21, 55). It can be proposed that an increase in endoplasmic reticulum ceramide might serve as a compensatory mechanism balancing the ceramide level in ASM−/− mice. However, a variety of additional lipidomic mechanisms determining alcohol consumption, which include several interconnected lipid species besides ceramide (17, 18), might be altered in Asm−/− mice. Thus, further analysis of possible compensatory pathways is needed to clarify the exact pathways.

The analysis of anxiety/depression in Asm−/− mice showed higher anxiety of these animals in the EPM, but not in the OF test. These differences might be related to the specific features of behavior investigated in these tests. Thus, the behavioral pattern of animals investigated in the OF test is mostly based on the exploratory behavior and locomotor activity. In contrast, cue-related behavior is mostly investigated in the EPM (56). It has been proposed that emotionality is a multi-dimensional parameter, which can be explored from various perspectives. Thus, different kinds of environments, e.g., open spaces, illuminated or elevated areas, might yield different kinds of behavioral responses (57).

Our behavioral data do not replicate the previous findings showing lower anxiety and depression levels in Asm−/− mice manifested in the higher center time in the OF test and time spent in the light compartment of the light-dark box, as well as shorter latency of first eating in the NSF test and shorter immobility time in the FST (21). However, the study by Gulbins et al. (21) was performed on the animals aging up to 7 weeks, which do not develop any behavioral manifestations of Niemann-Pick disease and are not characterized by the excessive sphingolipid accumulation in the tissues. In the present study we used 8–10-weeks old Asm−/− mice, which might be considered as a model of an early stage Niemann-Pick disease. Investigation of these animals showed that they do not possess full symptom expression yet, but have a slight impairment of locomotor activity in the OF test. However, the difference in the age might determine sphingolipid accumulation in the tissues, which might reverse the low depression and anxiety phenotype observed in younger ASM−/− mice. Moreover, the reason of this dissociation might be also based on the differences in the experimental conditions. In our experiment the animals received alcohol/saline injections 30 min before all tests, which can be considered as a mild stress. However, the present investigation also revealed that stress might affect animal behavior and even overwrite the Asm genotype-driven differences (21).

The data on the anxiety/depression state in Asm−/− mice are in line with the clinical studies on patients with Niemann-Pick disease. Niemann-Pick disease is a rare neurovisceral lysosomal storage disorder with a heterogeneous phenotype. The pathogenesis of types A and B of this disorder are based on Asm deficiency followed by sphingomyelin accumulation in the large, lipid-laden foam cells, which are present in the liver, spleen, lymph nodes, adrenal cortex, lung airways, and bone marrow (58). To our knowledge, no data on the anxiety and depression state of these patients are available. However, enhanced anxiety is observed in the patients with Niemann-Pick diseases, type C (59), which is characterized by sphingolipid accumulation in biological tissues and organs due to the mutations in the *NPC1* or *NPC2* genes (60). It was shown that ASM activity in the cells of these patients is also reduced (61). Moreover, BALB/cJ *Npc1* ^nih^ mice with *NPC1* haploinsufficiency, an animal model of type C Niemann-Pick disease, are characterized by increased anxiety-like behavior in early maturity. However, to our knowledge, the level of ASM activity in these animals was not measured (62). Thus, our data on the anxiety/depression state of Asm−/− mice, which might be considered as a model of Niemann-Pick disease type A and B, might add on to the current knowledge of the pathogenesis of this disorder.

The higher anxiety pattern in Asm−/− mice is in line with higher alcohol preference observed in these animals. Previous clinical data show that higher basal anxiety level might result in greater alcohol intake, which can promote alcohol abuse in humans (63). Moreover, a shared genetic risk for both anxiety and alcohol use disorder was observed in family studies (64, 65). Animal data also confirm these observations. For example, Spanagel et al. (66) showed that a high anxiety level in the EPM is associated with higher voluntary drinking in rats.

Interestingly, forced treatment with alcohol in a moderate dose (2 g/kg) had anxiolytic and depressogenic properties in Asm−/− mice. No such effects were observed in wt littermates. Anxiolytic effects of alcohol were manifested in an increase in the time spent, entries and locomotion in the open arms of the EPM. Depressogenic effects became evident by an increase in the floating time in the FST in Asm−/− mice. These data resemble our previous results on Asm+/- mice, which show only partial reduction of Asm activity in the brain. Similar anxiolytic and depressogenic effects of alcohol were observed in these animals (27). A reduction in anxiety level observed in homo- and heterozygous mice with reduced Asm activity are in line with literature. It was shown that rats preselected for high anxiety in the EPM demonstrate more pronounced anxiolytic effects of an alcohol injection compared to low anxiety animals (67). And vice versa, a rodent line preselected for high alcohol consumption showed stronger anxiolytic effects of alcohol in a battery of emotional tests (68, 69).

Similar depressogenic effects of alcohol, as it was shown in Asm−/− mice line, were also shown before (70, 71). A similar dissociation in the effects of alcohol on the emotional behavior was observed in our previous studies on mice with Asm overexpression (27). It should be noted that despite of the high comorbidity of anxiety and depression, certain pathways and neurological mechanisms of these disorders differ in the brain (72). Thus, our findings may suggest that Asm-mediated mechanisms might at least partly contribute to the pathways determining this difference, particularly after alcohol treatment. Moreover, the schedule of alcohol administration might mediate this dissociation. The EPM test was performed after two injections of alcohol, and, thus, the anxiolytic effects of alcohol might refer to its acute action. However, the FST was performed after a series of five alcohol injections referring to a rather subchronic alcohol treatment that caused depressogenic effects. Thus, it might be proposed that the duration of treatment might also affect alcohol action in Asm−/− mice.

Our previous data showed that a depressed state in animals with Asm overexpression can be partially reversed by voluntary alcohol consumption (27). Naïve Asm−/− mice are also characterized by higher alcohol preference and anxiety level. As alcohol treatment in these animals has anxiolytic effects, it might be speculated that Asm−/− mice might consume more alcohol compared to wt littermates in order to reduce their anxiety level. It is well-known that individuals use alcohol to enhance coping with stress and to reduce manifestations of mood disorders, especially depression, and anxiety. Chronic moderate alcohol drinking might reduce physiological and behavioral signs of anxiety and depression in humans (73). On another hand, anxiety and depression might enhance alcohol consumption and higher alcohol intake is associated with lower levels of nervousness (74). Thus, our data show that alcohol might be voluntarily used by ASM−/− mice to control their anxiety level. We believe that our study provide an additional confirmation of the hypothesis on the contribution of Asm to alcohol instrumentalization of emotional state, showing that alcohol might be used voluntarily to control possible emotional effects of the Niemann-Pick disorder.

Previously we observed that the reversion of a depressed state in mice with Asm overexpression with alcohol was mediated by partial normalization of Asm activity and downstream monoaminergic deficiency in the nucleus accumbens of these mice (27). Thus, it was proposed that alterations in sphingomyelin pathway of ceramide synthesis might mediate the effects of alcohol on emotional state (28). In the present study we checked if other enzymes of the sphingomyelinase pathway of ceramide metabolism contribute to the mechanisms of alcohol-induced decrease in anxiety and an increase in depression level in Asm−/− animals. The activities of Nsm, a sister enzyme of Asm involved in ceramide production, and Ac and Nc, which catalyze ceramide degradation to sphingosine, were analyzed. It was observed that voluntary alcohol consumption does not affect the activities of these enzymes neither in Asm−/− animals, nor in wt littermates. Thus, it can be suggested that no additional compensatory changes in the sphingomyelinase pathway of ceramide synthesis develop in these animals, confirming the specific contribution of Asm in the observed phenotypes.

Altogether, the present findings might add on to our hypothesis of an involvement of Asm in the mechanisms of comorbidity between alcoholism and anxiety and depression. We suggest that the lack of Asm might facilitate the anxiolytic and depressogenic effects of alcohol as well as an enhanced drinking pattern in mice. Further analysis of specific mechanisms of this comorbidity in Asm−/− mice is needed as the understanding of particular pathways might allow developing a new therapeutic strategy for comorbid psychiatric disorders as well as pathogenetic mechanisms of type A and B Niemann-Pick disease.

## Data Availability

The datasets generated for this study are available on request to the corresponding author.

## Ethics Statement

All experiments were carried out in accordance with the National Institutes of Health guidelines for the humane treatment of animals and the European Communities Council Directive (86/609/EEC) and were approved by the local governmental commission for animal health (Regierung von Mittelfranken).

## Author Contributions

LK, JK, EG, MR, and CPM initiated the study. LK, CM, VE, and MP performed the experiments. LK, VE, CPM, and CM analyzed the data. MR provided the animals. LK and CPM wrote the manuscript. All authors discussed the manuscript.

### Conflict of Interest Statement

The authors declare that the research was conducted in the absence of any commercial or financial relationships that could be construed as a potential conflict of interest.
